# Non-thermal resection device for residual Barrett ablation in patient already treated by endoscopic submucosal dissection for initial esophageal neoplasia with high grade dysplasia

**DOI:** 10.1055/a-2695-4078

**Published:** 2025-09-18

**Authors:** Antonio Ciccone, Elena Brandi, Jean Grimaldi, Jérôme Rivory, Elena De Cristofaro, Elodie Cesbron-Métivier, Mathieu Pioche

**Affiliations:** 1 Gastroenterology and Digestive Endoscopy Unit, AST Pesaro Urbino, San Salvatore Hospital, Pesaro, Italy; 2 Gastroenterology and Endoscopy Unit, Edouard Herriot Hospital, Hospices Civils de Lyon, Lyon, France; 3 9318 Gastroenterology Unit, University of Rome Tor Vergata, Rome, Italy; 4 26966 Hepatogastroenterology Department, Angers University Hospital, Angers, France

Endoscopic treatment of early Barrett’s neoplasia has been established as a two-step approach 1 . In the first step, endoscopic resection for all visible neoplasia is performed. Endoscopic submucosal dissection (ESD) is globally accepted as a treatment for visible lesions, enabling a high rate of en bloc R0 resection 2 . In the second step, the ablation of all residual metaplasia is necessary to avoid the recurrence of neoplasia 1 . Thermal radiofrequency ablation (RFA) is currently used for this indication and obtains a significantly higher rate of complete eradication of metaplasia 3 . Despite RFA’s success, however, there is a subset of patients in whom complete eradication of metaplasia cannot be achieved 3 . Recently some non-thermal procedures were proposed. Our device is a powered non-thermal resection device 3 4 5 associating a rotative blade with suction.

We aimed to assess the efficacy and safety of a new non-thermal resection device for the eradication of residual Barrett’s neoplasia after ESD. In addition, the adverse events rate, during the procedure and during the follow-up period, was measured.

We report on the use of a non-thermal resection device system ( Fig. 1 ) for complete ablation of residual Barrett’s esophagus ( Video 1 ). A 65-year-old woman had already undergone esophageal ESD for a 20 × 15-mm nodular lesion with high grade dysplasia that originated on C1M3 Barrettʼs esophagus. Due to residual low grade dysplasia on the margins, the patient was subsequently included in a randomized protocol (endo-Barrett) comparing the non-thermal resection device with radiofrequency to destroy the residual Barrett’s tissue. Therefore, the ablation of the residual Barrett’s tissue was performed with this ablation tool three months after the ESD procedure. The complete destruction of residual tissue was achieved without any adverse event. The resected tissue was recovered in a dedicated specimen trap. No esophageal strictures developed during the follow-up period.

**Fig. 1:**
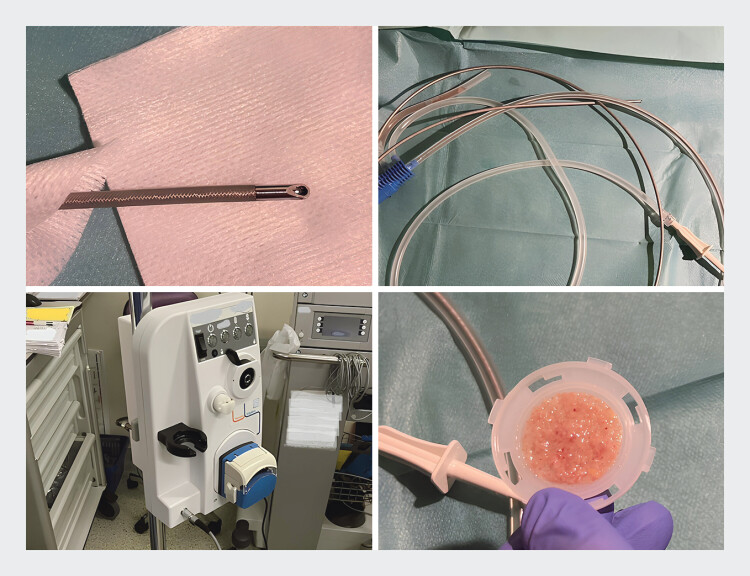
The non-thermal endoscopic powered resection device with its tip, catheter, console, and dedicated specimen trap for tissue recovery.

Use of the new non-thermal resection device for complete ablation of residual Barrett in patient previously treated with esophageal endoscopic submucosal dissection for HGD lesion.Video 1

The great capacity to destroy the tissue allows a total avulsion of the Barrett’s esophagus without any adverse event. The non-thermal resection device system may be a promising device for complete ablation of residual dysplastic Barrett’s esophagus after endoscopic resection if safety and effectiveness to prevent recurrence is further demonstrated.

Endoscopy_UCTN_Code_TTT_1AO_2AG

## Notes

Endoscopy E-Videos https://eref.thieme.de/e-videos
*E-Videos* is an open access online section of the journal *Endoscopy* , reporting on interesting cases and new techniques in gastroenterological endoscopy. All papers include a high-quality video and are published with a Creative Commons CC-BY license. Endoscopy E-Videos qualify for HINARI discounts and waivers and eligibility is automatically checked during the submission process. We grant 100% waivers to articles whose corresponding authors are based in Group A countries and 50% waivers to those who are based in Group B countries as classified by Research4Life (see: https://www.research4life.org/access/eligibility/ ). This section has its own submission website at https://mc.manuscriptcentral.com/e-videos .
